# Transcatheter embolization as a rescue therapy for refractory pelvic kaposiform hemangioendothelioma with Kasabachi–Merritt phenomenon: a case report

**DOI:** 10.3389/fped.2026.1821994

**Published:** 2026-09-03

**Authors:** Yi Xiong, Qi Di, Sanlin Li, Jiajie Cao, Chenghao Chen, Gang Shen

**Affiliations:** Hemangioma and Vascular Anomaly Center, Capital Center for Children’s Health, Capital Institute of Pediatrics, Capital Medical University, Beijing, China

**Keywords:** kaposiform hemangioendothelioma, Kasabach–Merritt phenomenon, pediatric, sirolimus, transarterial embolization, vascular anomaly

## Abstract

Kasabach–Merritt phenomenon (KMP) is a life-threatening consumptive coagulopathy in children with kaposiform hemangioendothelioma (KHE) or tufted angioma. Until now, there has been no well-accepted treatment regimen in the literature. A pelvic KHE is very rare and its deep anatomical location and invasive growth pattern toward adjacent vital organs pose significant management challenges. Here, we report a 10-month-old girl presenting with KMP secondary to a pelvic KHE. Initial systemic therapy with sirolimus and corticosteroids failed to control her KMP. Transcatheter arterial embolization was performed using polyvinyl alcohol particles and coils. After embolization, the tumor’s blood supply decreased significantly and the patient’s platelet counts and coagulation markers returned to normal in 3 days. At the 2-year follow-up, her coagulation function remained normal. The perineal cutaneous rash resolved and the tumor’s volume decreased substantially. This case demonstrates that transarterial embolization can be an effective therapeutic choice when systemic treatment has failed.

## 1 Introduction

Kasabach–Merritt phenomenon (KMP) is a life-threatening complication characterized by thrombocytopenia and consumptive coagulopathy. It occurs in approximately 40.3%–64.3% of children diagnosed with kaposiform hemangioendotheliomas (KHEs) (1–3). The associated mortality rate ranges from 30% to 40% (4). The International Society for the Study of Vascular Anomalies categorizes KHE as a locally invasive vascular neoplasm. Although KHEs most commonly occur in cutaneous and subcutaneous tissues, they may also develop in deep anatomical sites, including the retroperitoneum, bone, and skeletal muscle.

Current guidelines recommend that sirolimus combined with corticosteroids be the first-line therapy for complicated KHE/KMP (5). Nevertheless, children with critical KMP may experience rapid clinical deterioration before systemic treatment achieves full therapeutic effects. Therefore, arterial embolization may be needed as an adjunctive treatment.

A pelvic KHE is extremely rare in the literature. Here, we report a girl with a pelvic KHE associated with KMP who was successfully treated with transarterial embolization combined with sirolimus and corticosteroids. This case highlights that transarterial embolization can be an effective therapeutic choice when systemic treatment has failed.

## 2 Case report

**Figure 1:**
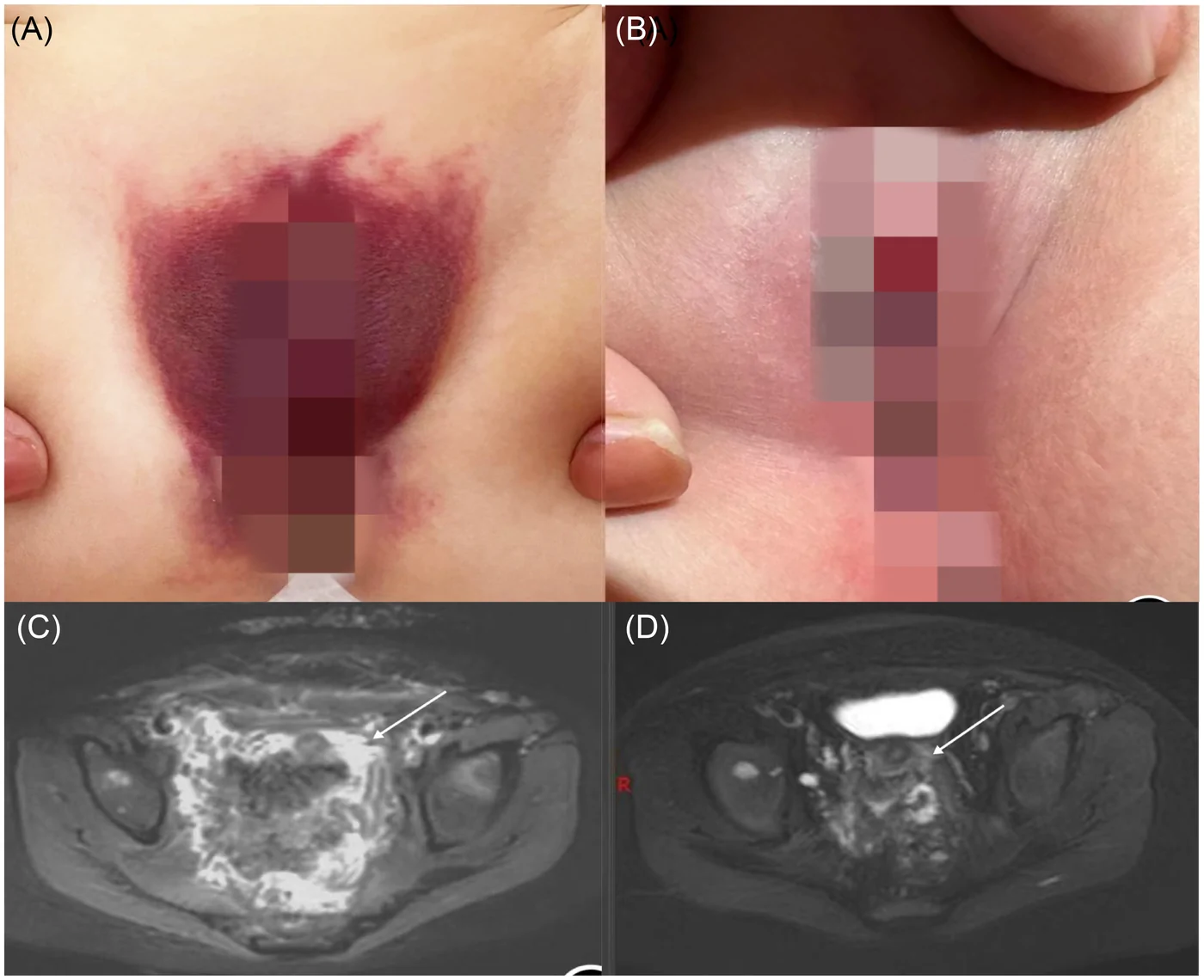
Perineal rash and pelvic magnetic resonance imaging (T2-weighted) before and after treatment. **(A)** Purple indurated perineal lesion. **(B)** Complete resolution of the rash after treatment. **(C)** Hyperintense pelvic mass behind the uterus with bone invasion. **(D)** Significant reduction in tumor volume after treatment.

A 10-month-old girl was admitted to our hospital with a progressively darkened purple indurated perineal lesion with ill-defined borders (Figure 1A). Physical examination revealed scattered petechiae on the abdominal wall. Initial laboratory tests showed severe thrombocytopenia (17 × 10^9^/L; normal 191–516 × 10^9^/L), hypofibrinogenemia (0.24 g/L; normal 2.0–4.0 g/L), a markedly elevated D-dimer level (83.44 mg/L FEU; normal 0–0.55 mg/L FEU), prolonged prothrombin time of 14.5 s (normal 0–13.0 s), and prolonged activated partial thromboplastin time of 35.2 s (normal 22.3–32.5 s).

Pelvic magnetic resonance imaging revealed a 35 mm × 44 mm × 67 mm hyperintense mass on T2-weighted images behind the uterus, with adjacent bone invasion (Figure 1C).

**Figure 2:**
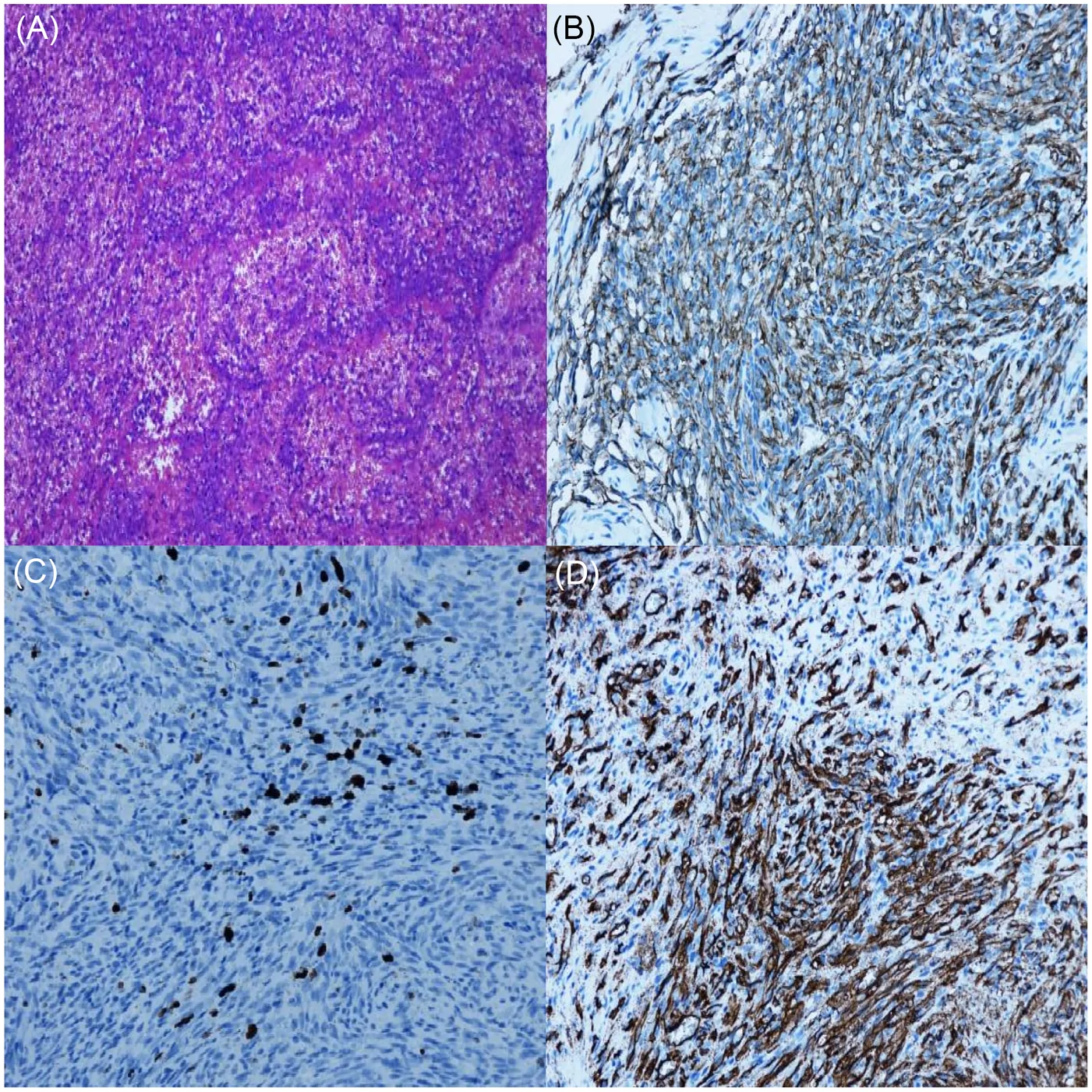
Histopathological features of the KHE. **(A)** HE staining (×100) shows nested and lobular short spindle-shaped tumor cells, accompanied by hemorrhage and degeneration. **(B)** CD31 positive (×200). **(C)** Ki-67 staining (×200). **(D)** CD34 positive (×200).

After a transfusion of platelets, fresh frozen plasma, and fibrinogen (FIB) concentrate, the patient's coagulation parameters improved, with a FIB level of 1.51 g/L and platelet count of 312 × 10^9^/L. Due to the mass’s deep location and hypervascularity, a percutaneous biopsy would not obtain enough specimens, so exploratory laparotomy was performed to evaluate the extent of the locally invasive pelvic tumor and obtain a histopathological diagnosis. During the operation, the tumor was found to be unresectable due to extensive local invasion. The biopsy confirmed a KHE, with positive staining for CD31 (platelet endothelial cell adhesion molecule-1), CD34 (hematopoietic progenitor cell antigen), D2-40 (Podoplanin), and ERG (ETS-related gene); a low Ki- 67 index (10%); and negative staining for GLUT1 (glucose transporter 1), F8 (factor-VIII- related antigen), AE1/AE3 (cytokeratin AE1/AE3), and Desmin (Figure 2).

**Figure 3:**
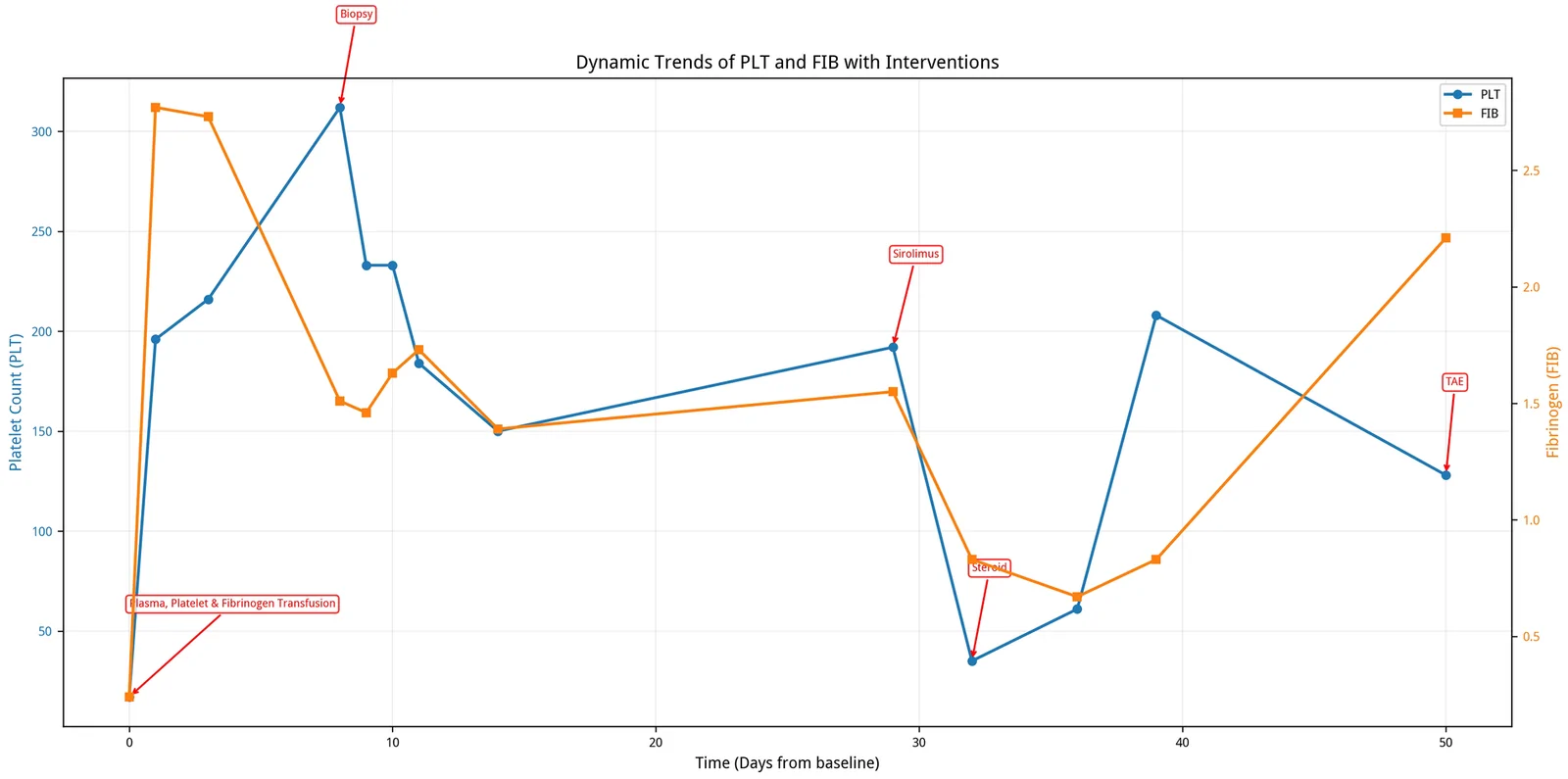
The trends in platelet and fibrinogen levels prior to transcatheter embolization therapy.

Systemic treatment was attempted first. Intravenous methylprednisolone at a dose of 2 mg/kg/day and oral sirolimus at a dose of 0.8 mg/m^2^ twice daily were initiated. Sirolimus was titrated to maintain a trough concentration of 5–10 ng/mL (5, 6). After 20 days of systemic therapy, thrombocytopenia and coagulopathy persisted; thus, transarterial embolization was planned to correct the coagulopathy. FIB and platelet trends are shown in Figure 3.

**Figure 4:**
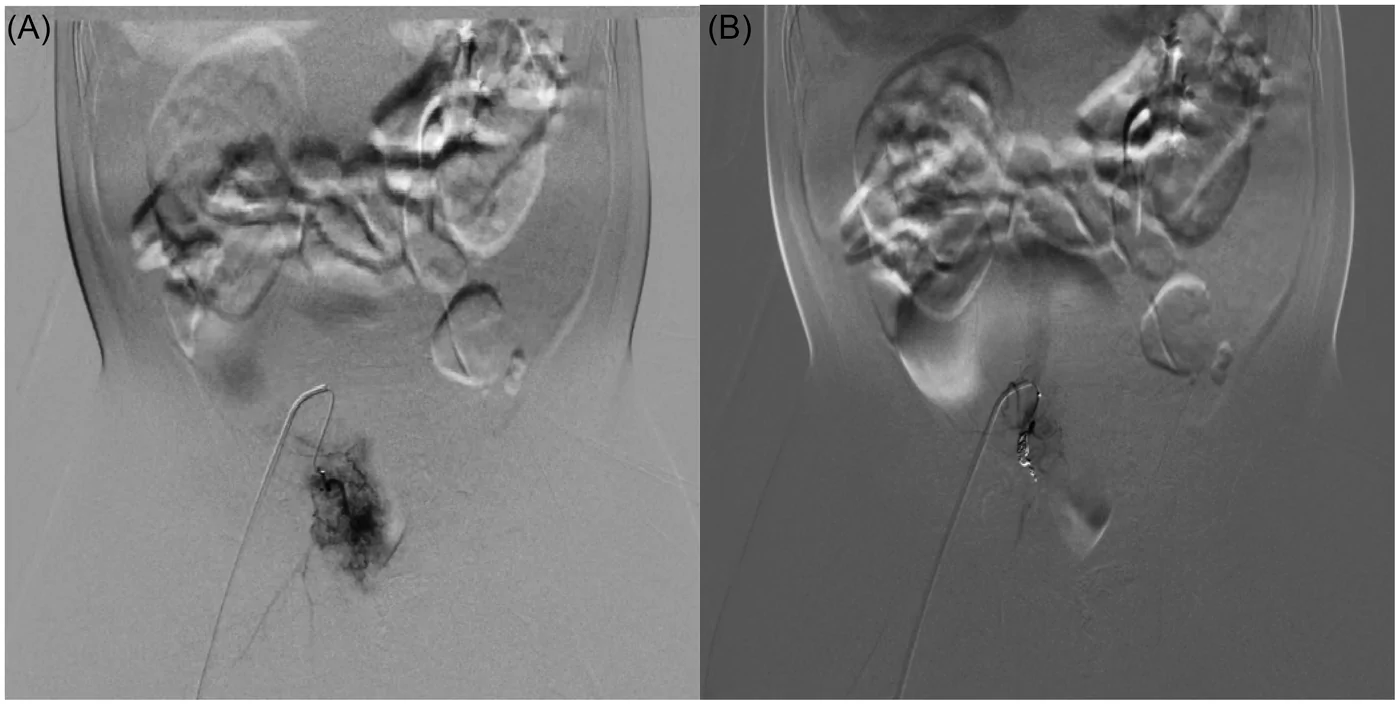
Pelvic angiography. **(A)** Before embolization: tumor staining supplied by branches of the right internal iliac artery. **(B)** After embolization: markedly diminished tumor staining.

Under general anesthesia, percutaneous access to the right femoral artery was established using a 4-Fr sheath. Pelvic angiography was performed with a 4-Fr Cobra catheter. A hypervascular tumor nourished by multiple small branches originating from the right internal iliac artery was revealed (Figure 4A). A 1.9-Fr microcatheter was advanced to achieve superselective cannulation of tumor-feeding vessels, in order to avoid extensive embolization of the internal iliac artery branches. Real-time angiography was performed continuously throughout the injection of the embolic agent to monitor blood flow status strictly and rule out embolus reflux. The injection was stopped once blood flow velocity obviously decreased to prevent non-target embolization. Targeted embolization was performed with 300–500 μm polyvinyl alcohol particles, and coils (3 mm × 6 cm × 2, 4 mm × 8 cm × 2) were additionally deployed to occlude the dominant feeding branch. The embolization was immediately terminated when tumor staining was markedly diminished (Figure 4B). Since our primary goal was to correct refractory consumptive coagulopathy, only a partial embolization was performed.

No postoperative adverse symptoms, including abdominal pain, gluteal soreness, or bowel and bladder dysfunction, were observed in the patient. Three days after embolization, her platelet count increased to 242 × 10^9^/L, her fibrinogen level increased to 2.03 g/L, her D-dimer level decreased to 3.4 mg/L FEU, and her prothrombin time and activated partial thromboplastin time returned to the normal range. Postoperative pelvic MRI confirmed no evidence of ischemic injury to adjacent pelvic organs.

After discharge, corticosteroids were gradually tapered as planned, and long-term oral sirolimus maintenance therapy was initiated to prevent disease recurrence. Regular follow-up was performed, including complete blood counts, coagulation function, lipid profiles, renal function, hepatic function, and sirolimus trough levels. No treatment-related adverse events (including oral mucositis, diarrhea, hyperlipidemia, or immunosuppression-related infections) were observed throughout the entire course of treatment.

At the 2-year follow-up, the patient remained on daily oral sirolimus therapy, with normalized coagulation function, complete resolution of cutaneous rashes (Figure 1B), and significant regression of the vascular lesion, confirmed by magnetic resonance imaging (Figure 1D). She continues on maintenance sirolimus therapy and attends regular follow-up appointments at our department.

## 3 Discussion

KMP was first characterized by Kasabach and Merritt in 1940 (7). It is defined as severe consumptive coagulopathy, with substantial mortality risk (8). KHE is a locally aggressive vascular neoplasm that is pathologically distinct from common infantile hemangioma (9).

The clinical management of KMP remains challenging. Current treatment modalities include sirolimus, corticosteroids, vincristine, interferon, surgical resection, and transarterial embolization. Sirolimus-centered regimens have become the first-line therapy for KHE/KMP (5, 10–13). A previous randomized controlled trial confirmed that sirolimus combined with prednisolone achieves better control of platelet and fibrinogen levels compared with sirolimus monotherapy, with fewer transfusion requirements and disease sequelae (13). Consensus guidelines similarly recommend combined systemic treatment as the standard care (5). For patients with refractory KHE/KMP unresponsive to first-line sirolimus-corticosteroid therapy, sirolimus combined with aspirin is a promising targeted salvage regimen (14). Aspirin reduces intratumoral platelet sequestration and alleviates consumptive coagulopathy, while sirolimus exerts antiangiogenic activity via mTOR pathway inhibition. However, systemic medical therapy typically requires a relatively long time to reach therapeutic efficacy. Patients with KMP may require urgent interventional treatment to prevent fatal hemorrhage. Transarterial embolization reduces tumor perfusion and intralesional platelet trapping, thereby rapidly reversing coagulation dysfunction (15–18). Brill et al. reported that transarterial embolization combined with sirolimus therapy accelerates KMP resolution. Patients who received combined treatment achieved hematological recovery within a median of 7 days, compared with 3 months in patients treated with sirolimus alone (19). Accordingly, transarterial embolization can be used as a life-saving bridging intervention that stabilizes patients until systemic medications take full effect.

Our patient exhibited several distinctive clinical characteristics. First, a pelvic KHE is extremely rare, and surgical resection was infeasible due to its deep pelvic location and invasive infiltration of adjacent vital structures. Second, the patient developed progressive KMP despite combined first-line systemic therapy, which required additional adjuvant interventional treatment. Furthermore, transarterial embolization induced rapid, durable hematological recovery, with sustained disease improvement observed at the 2-year follow-up.

## 4 Conclusions

A pelvic KHE complicated by KMP is a rare, life-threatening clinical entity. When combination systemic therapy with sirolimus and corticosteroids fails to control severe coagulopathy, transarterial embolization is a safe and effective salvage intervention. The combination of transarterial embolization and systemic medication enables rapid hematological recovery and durable long-term disease control. This integrated therapeutic approach should be considered for patients with refractory, deep-seated KHE complicated by KMP.

## Data Availability

The raw data supporting the conclusions of this article will be made available by the authors, without undue reservation.
